# A multi-intent based multi-policy relay contrastive learning for sequential recommendation

**DOI:** 10.7717/peerj-cs.1088

**Published:** 2022-08-31

**Authors:** Weiqiang Di

**Affiliations:** School of Computer and Information Technology, Beijing Jiaotong University, Beijing, Beijing, China

**Keywords:** Sequential recommendation, Contrastive learning

## Abstract

Sequential recommendations have become a trending study for their ability to capture dynamic user preference. However, when dealing with sparse data, they still fall short of expectations. The recent contrastive learning (CL) has shown potential in mitigating the issue of data sparsity. Many item representations are destined to be poorly learned due to data sparsity. It is better to pay more attention to learn a set of influential latent intents that have greater impacts on the sequence evolution. In this article, we devise a novel multi-intent self-attention module, which modifies the self-attention mechanism to break down the user behavior sequences to multiple latent intents that identify the different tastes and inclinations of users. In addition to the above change in the model architecture, we also extend in dealing with multiple contrastive tasks. Specifically, some data augmentations in CL can be very different. Together they cannot cooperate well, and may stumbling over each other. To solve this problem, we propose a multi-policy relay training strategy, which divides the training into multiple stages based on the number of data augmentations. In each stage we optimize the relay to the best on the basis of the previous stage. This can combine the advantage of different schemes and make the best use of them. Experiments on four public recommendation datasets demonstrate the superiority of our model.

## Introduction

With the development of Internet services, recommendation systems have been extensively applied in online platforms to alleviate information overload in fields like e-commerce, music streaming and news portals. Accurate recommendation can greatly improve the interactive experience of users. Due to the sequential signals underlying user behavior, it is essential to learn user’s dynamic preference by modeling the sequential dependency over the user-item interactions. Sequential recommendation sorts items by the timestamp and captures useful sequential patterns from users’ historical behaviors to predict items that users may be interested in.

Sequential recommendation has attracted increasing attention recently. Various models have been proposed and have achieved promising results. The Markov chain (Zimdars, Chickering & Meek, 2013) adopts K-order user-item interaction sequential transitions. GRU4Rec (Hidasi et al., 2015) applies recurrent neural networks (RNN) to model the sequential dependency for recommendation. Works like Caser and NextItNet (Tang & Wang, 2018; Yuan et al., 2019) have attempted to employ convolutional neural networks (CNN) to capture the sequential dependency with a flexible order. Subsequently, self-attention mechanisms (Kang & McAuley, 2018; Vaswani et al., 2017) attend to information at different positions from different representation subspaces to retain long-term information. Recently, Multi-Interest Network with Dynamic Routing (MIND) (Li et al., 2019) employs dynamic routing to transform history behaviors into the user representations (Sabour, Frosst & Hinton, 2017), which can reflect users’ different intents. ComiRec (Cen et al., 2020) uses a self-attention mechanism and dynamic routing to capture multiple intents from the interaction sequences. DSS (Ma et al., 2020) proposes a seq2seq training strategy and exploits supervision signals by investigating the long term behavior instead of just the next immediate item. ISRec (Li et al., 2021) adopts an intention graph to capture the correlations among user intentions. ICLRec (Chen et al., 2022) first captures users’ intents from behavior sequences *via* clustering and then uses the contrastive task to maximizes the alignment between a view of sequence and its corresponding intent.

Though they have achieved promising results, current model learning mainly relies on observable user-item interactions. This prevents them from reaching full potential due to the problem of data sparsity as deep neural models are inherently data-hungry while most users only interact with a small portion of enormous items. The recently emerging contrastive learning (CL) methods (Jaiswal et al., 2021; Liu et al., 2021) are designed which can reduce the reliance on data labels. The essential idea behind CL is to extract informative knowledge from unlabeled data through well-designed self-supervised tasks. CL consists of two core components: data augmentation and contrastive loss. The common data augmentations in current CL-based sequential models are three random augmentation operators item masking, item cropping, item reordering used in CL4SRec (Xie et al., 2020) and the semantic augmentation proposed in DuoRec (Qiu et al., 2022). As for the contrastive loss, they treat it as a supplementary and leverage a multi-task learning strategy to jointly train the traditional ranking loss and proposed contrastive loss. The mainstream CL-based sequential models adopt the self-attention module as the backbone network, which is used to encode the original/augmented sequences and learn user representations for the global-level contrast and prediction.

**Figure 1:**
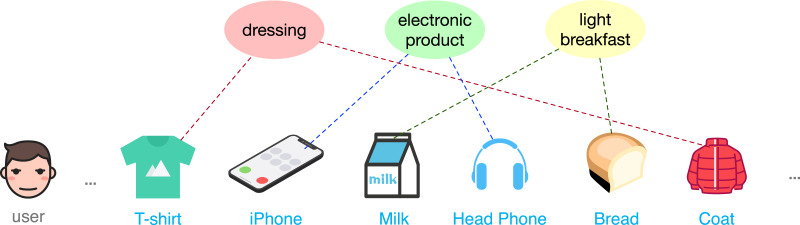
An illustration of multiple intents in a user sequence.

Despite effectiveness, the traditional self-attention module may not be very suitable as the backbone in the CL-based sequential models. It neglects the fact that users often have multiple intents in terms of different themes of items over a periods of time, which however is an important feature in the recommendation domain. It is the latent intents that drive a user’s interactive behavior and guide the evolution of sequences as shown in Fig. 1. However, due to data sparsity, the expressiveness of many learned item embeddings will be limited. Shifting the focus from learning each item representations to learning a small number of more general intent representations will alleviate this challenge. A latent intent is usually associated with many items, and richer information makes its representation easier to learn. For the above two considerations, we adapt the self-attention module to be intent-aware in this article. Specifically, we group users’ historical behaviors into several latent intents, and the updated representation for each item will be intent-aware by being reconstructed from these latent intents. Another challenge comes from the exploitation of multiple contrastive tasks, especially those with very different underlying mechanisms. For example, semantic augmentation does not fit easily with the operators in random augmentation. In order to make better use of them, we propose a multi-policy relay training strategy. It divides the model learning into multiple stages, where each stage corresponds to a CL task with one data augmentation operator. Through multiple relay training, interference among CL tasks can be relieved, and ultimately lead to better representation learning.

The main contributions of this article are:

- We adapt the self-attention module to capture latent intents that are key affecting users’ interactive behaviors in the recommendation field and shift from the focus of learning each item representations to learning a small number of more influential latent intents.
- We propose a multi-policy relay training strategy to collaborate multiple contrast tasks that have very different underlying mechanisms and make them work better together.
- Extensive experiments are conducted on four public datasets, demonstrating that our approach is consistently better than a number of competitive baselines.

## Related work

Our work bridges three areas of research: sequential recommendation, multi-intent recommendation and contrastive recommendation. We therefore mainly review the most related articles in these areas.

### Sequential recommendation

In real-world scenarios, the user’s next behavior not only relies on the static long-term preference, but also depends on the current intent to a large extent, which might be inferred by a small set of the most recent interactions. Sequential recommendation (SR) relies on users’ recent behavior to make decisions (Rendle, Freudenthaler & Schmidt-Thieme, 2010; Lv et al., 2019). As recurrent neural networks (RNN) can well capture the dependencies among items in natural language processing (NLP). RNN-based models are introduced in the sequential recommendation. GRU4Rec (Hidasi et al., 2015) is the first work to employ RNN in sequential recommendation. However, RNN has some shortcomings, such as difficulty in capturing long-term dependency, poor parallelism, and too strict order assumptions for interaction sequence. Subsequently, some convolutional neural networks (CNN) were proposed and obtained good results (Tang & Wang, 2018; Yuan et al., 2019). It is suitable to capture the dependency relationship across local items. One problem of CNN-based models is that they have difficulty in capturing relations between items that are not nearby. Recently, there are works that employ advanced techniques, *e.g*., attention mechanisms (Pei et al., 2017; Mei et al., 2018; Ying et al., 2018; He et al., 2021) and gating mechanisms (Ma, Kang & Liu, 2019) for sequential recommendation to model the importance of different items in the input sequence. Typically, SASRec (Kang & McAuley, 2018) utilizes self-attention to exploit the mutual influence between historical interactions.

### Multi-intent recommendation

The main assumption of current recommendation models is that people sharing similar interactions in history tend to have similar preferences in the future (Koren, Bell & Volinsky, 2009). It does have demonstrated its remarkable ability in improving the recommendation performance. In reality, users can interact the same item with multiple latent intents, which is however ignored. Suppose such a scenario, there are three people ${S_1}$, ${S_2}$, ${S_3}$ and their movie viewing history: ${S_1}$ = {Movie 1, Movie 2}, ${S_2}$ = {Movie 2, Movie 3}, ${S_3}$ = {Movie 2, Movie 4}. Our task is to predict whether user ${S_1}$ prefers to watch Movie 3 or 4 next. Without other information this would be hard to guess since both user 2 and user 3 watched Movie 2 at the same time. But if we know that user ${S_1}$ and ${S_3}$ like to watch sci-fi movies while user ${S_2}$ prefers action movies, we may find that Movie 4 is a better recommendation because it has been viewed by a sci-fi oriented user. User intent modeling has recently gained attention for its capability to capture dynamic user intents based on historical interaction sequence (Liu et al., 2020; Pan et al., 2020; Tanjim et al., 2020). Multi-Intent Translation Graph Neural Network (MITGNN) (Liu et al., 2020) combines a translation-based model with a GNN model to learn users’ multiple intents, where the intents are embedded in different translations. MIND (Sabour, Frosst & Hinton, 2017) and ComiRec (Cen et al., 2020) mainly focus on capturing multiple intents by utilizing dynamic routing from users’ behavior sequences to reflect recommendation diversity. DSS (Ma et al., 2020) change the traditional sequence-to-item training strategy to the sequence-to-sequence strategy based on latent self-supervision and disentanglement. ISRec (Li et al., 2021) propose a structured intention-aware model which first discover user intentions from their past behaviors and then adopt an intention graph to capture the correlations among user intentions. ICLRec (Chen et al., 2022) employs clustering to extract users’ intent distributions from users’ behavior sequences. And it integrates the intents captured into the sequential model using a contrastive SSL loss.

### Contrastive recommendation

Data acquisition is difficult since personalized recommendation relies on the data generated by users themselves, while most users only interact with a small portion of numerous items. Furthermore, the prediction loss dominates the model learning, which neglects the intrinsic structure of the context data. Without overcoming the above problems, it is challenging to further improve the performance. Self-supervised learning (SSL) (Liu et al., 2021), a new learning paradigm that can reduce the reliance on data labels, recently has received considerable attention. The key of SSL is to mine information from unlabeled data *via* well-designed self-supervised tasks based on the understanding of recommendation domain knowledge. Contrastive learning (CL) (Jaiswal et al., 2021; Liu et al., 2021) has become the dominant branch in SSR. It obtains supervision signals from the data itself and usually predicts part of the data from other parts. CL treats every instance as a class, and then pulls views of similar instances closer in the embedding space, and pushes views of different instances apart, where the views are created by imposing different transformations on the original data. Deep InfoMax (Hjelm et al., 2018) first employs a contrastive learning task to learn the mutual information between a local block and its global context. CPC (Van den Oord, Li & Vinyals, 2018) then proposes a scheme which predicts the future in latent space by using a powerful autoregressive model. Inspired by the success of CL in other fields, researchers begin to explore possible application directions in recommender systems. Exploiting CL in sequential recommendation is still a new area of research with only a few works published thus far. To capture the intrinsic data correlations, S3Rec (Zhou et al., 2020) integrates SSL in sequential recommendation with four self-supervised optimization objectives to maximize the mutual information between item-attribute, sequence-item, sequence-attribute and sequence-sequence. CL4SRec (Xie et al., 2020) utilizes three random data augmentation operators (*i.e*., item cropping, item masking and item reordering), where two operators are randomly sampled and applied to each user sequence. DuoRec (Qiu et al., 2022) proposes the semantic augmentation operator which uses the target item as the supervision signal.

## Preliminaries

### Problem formulation

We first formulate the task of sequential recommendation here. It is referred to a model which takes a user’s behavior trajectories as input, and then processes with a well-designed algorithm to recommend appropriate items to the user. Suppose *V* is the item set, the interaction sequence of a user are constructed in chronological order $s = \left( {{v_1},{v_2}, \ldots ,{v_t}} \right)$, where ${v_i} \in V,0 \le i \le t$ describes the chronological order of item and *t* is the length of the sequence. With the definitions above, the problem can be formally defined as follows: Given a user’s historical item sequence *s*, our aim is to predict the next item that the user is likely to interact with at time step *t* + 1.

### Sequence-based data augmentation operators

**Figure 2:**
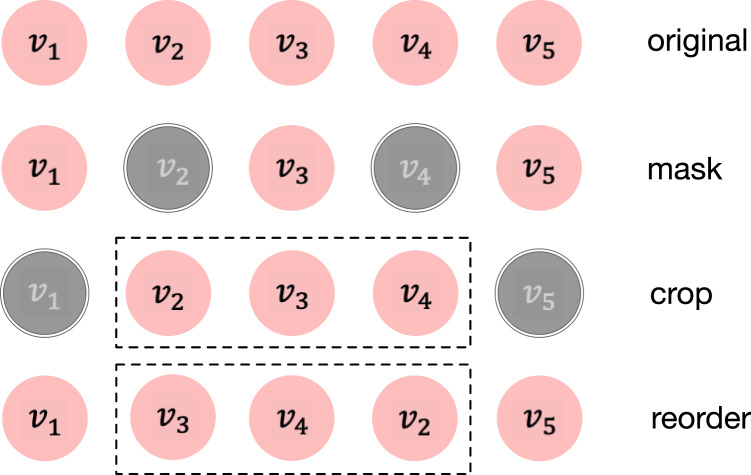
Three augmentation operators are adopted in this article. The original sequence is $s= {(v_1, v_2, v_3, v_4, v_5)}$. Grey elements mean they are not included in the newly generated sequence.

Previous methods in many domains on SSL have showed that data augmentations play an important role in improving the model performance (Ding et al., 2022; Li, Hou & Che, 2022; Tian et al., 2020). We now introduce the data augmentation methods in contrastive learning that will be used later. It should be noted that, in the Multi-Policy Relay part of our model, the more data augmentation methods used, the greater the time consumption, so we only select two of the three random augmentations as examples. Given an item sequence $s = ({v_1},{v_2},...,{v_t})$ which is a user’s behavior interactions, the sequence-based data augmentation operators $\tilde s = T(s)$ are listed here. Simple illustrations of those methods are also demonstrated in Fig. 2.

**Item masking.** Similar to Masked LM in BERT (Devlin et al., 2018). Item masking randomly masks a portion of items and replaces them using a specific token. The underlying assumption is that the intent that drives a series of interactions is relatively stable within an appropriate time period. Although the sequence becomes incomplete, the intent remains largely unaffected. Let *M* be the set containing the indices of the masked items, which is randomly drawn from the sequence with probability $\gamma$. The formal description of item masking is as follows:

(1) $$\matrix{ {{\rm }\tilde s = {T_{{\rm masking}}}(s) = ({{\tilde v}_1},{{\tilde v}_2},...,{{\tilde v}_t}),where\;{{\tilde v}_t}} \hfill & { = \left\{ {\matrix{ {{v_t},} \hfill & {t\ \notin\ M} \hfill \cr {{\rm mask},} \hfill & {t \in M} \hfill \cr } } \right.} \hfill \cr }$$

**Item cropping.** Analogous to the commonly used data augmentation technique image cropping in computer vision, item cropping randomly reserves a consecutive sub-sequence with length ${L_{\rm c}} = \lfloor\,\eta *|S|\rfloor$, where $\eta \in (0,1)$ controls the cropping length. This data augmentation operator can be formulated as:

(2) $$\tilde S = {T_{{\rm cropping}}}(S) = ({\tilde v_c},{\tilde v_{c + 1}}, \ldots ,{\tilde v_{c + {L_c} - 1}})$$

where *c* is the start index of the selected sub-sequence. This operator provides a way to infer the global view from a local view, which helps to learn more general representations.

**Semantic augmentation.** It is reasonable to think that different behavior sequences may imply the same user preference if they have the same target and we infer these sequences are semantically similar. Therefore, given two different user sequences ${s_i} = \left( {{v_{i,1}},{v_{i,2}}, \ldots ,{v_{i,{t^i}}}} \right)$ and ${s_j} = \left( {{v_{j,1}},{v_{j,2}}, \ldots ,{v_{j,{t^j}}}} \right)$, if the next item of ${s_i}$ and ${s_j}$, *i.e*., ${v_{i,{t^i} + 1}}$ and ${v_{j,{t^j} + 1}}$, are the same item, ${s_i}$ and ${s_j}$ are thought to be semantically similar.

### Noise contrastive estimation

Contrastive learning is one branch of self-supervised learning that dedicates to distinguish between similar data samples and dissimilar ones. After encoded by the model, inputs are transformed into a low-dimensional space so that samples that are similar will be close to each other and far away from the others in the representation space. Similar samples can be created by applying different transformations on the same data, or by gathering semantically similar data from different data. Generally, views of the same data are thought a positive pair, and views of different data are classified as negative pairs. It is worth noting that positive transformations should not introduce essential torture from each other. For example, the original semantics should not be changed after the positive transformation.

We adopt the commonly used noise contrastive estimation (NCE) (Gutmann & Hyvärinen, 2012) loss as the objective of contrastive tasks. Let *x* and ${x^ + }$ represent two similar data samples. Besides, we have a number of negative samples $x_i^ -$ that are dissimilar with *x*. We then have the loss function defined as:

(3) $${\ell _{{\rm NCE}}} = {\rm {\mathbb E}}\left[ { - \log \displaystyle{{{e^{f{{({x^ + })}^{\rm \top }}f(x)/\tau }}} \over {{e^{f{{({x^ + })}^{\rm \top }}f(x)/\tau }} + \sum\nolimits_i {{e^{f{{(x_i^ - )}^{\rm \top }}f(x)/\tau }}} }}} \right],$$

where $\tau$ is the softmax temperature parameter and f denotes the encoder our model represents.

## Methodology

In this section, we first give an overview of the proposed Multi-Intent based Multi-Policy Relay Contrastive Learning (MMRec) framework. Then, we mainly present the technical details of two core modules, which extends previous models from the model architecture and training strategy of combining multiple sequence-based augmentations in CL.

### Model overview

**Figure 3:**
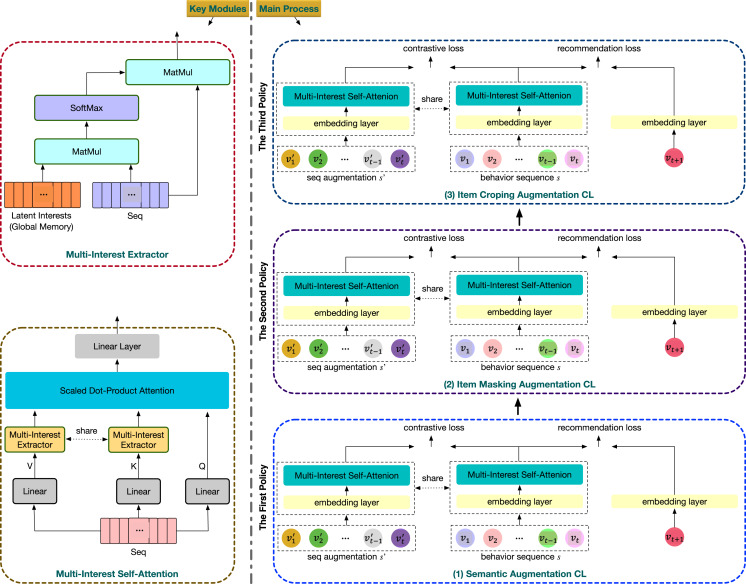
Overall framework of our proposed MMRec. The input is the user’s behavior sequence.

The overall structure of MMRec is illustrated in Fig. 3. All components are mainly developed around two innovations. The first is that MMRec integrates the multi-intent feature into the powerful self-attention module. The second is that MMRec divides the model training into multiple stages and each stage corresponds to a CL task, which can collaborate contrast tasks having very different underlying mechanisms. Let’s now trace the processing trajectory of a data sample. Suppose there are three different data augmentation operators ${D_s} = \{ {D_1},{D_2},{D_3}\}$ First we start the first stage of training by selecting ${D_1}$ from ${D_s}$. Given an input consisting a pair of similar behavior sequences generated from this data augmentation operator and a number of dissimilar samples, we get their embedding vectors *via* the embedding layer. The embeddings obtained will then be encoded by the upgraded self-attention module. Then we use the resulting sequence representation to compute the loss and update model’s trainable parameters. Now the first stage of training is over, and then we choose ${D_2}$ to start the second stage of training and so on.

### Embedding layer

As shown in Fig. 3, the input of MMRec is a historical sequence $s = ({v_1},{v_2},..,{v_t})$. If in training, there will also be an accompanying similar sample $s{\rm '} = (v_1^{'},\,\,v_2^{'},..,\,v_t^{'})$ and some dissimilar sequences. We convert the sequence into a fixed-length sequence $s = ({v_1},{v_2},..,{v_n})$ to facilitate network processing, where n represents the maximum sequence length we adopted. The sequence is truncated and only taken the recent *n* items if too long, otherwise, the sequence is padded to length *n*.

We construct an embedding matrix of items ${V} \in {{\mathbb R}^{(|V| + 1) \times d}}$, where *d* is the dimension of embedding. For an input $s = ({v_1},{v_2}, \ldots ,{v_n})$, we can obtain its embedding:

(4) $$E = ({{\bf e}_1},...,{{\bf e}_n}) \in {{\mathbb R}^{n \times d}}$$

where ${{\bf e}_i} \in {{\mathbb R}^d}$ is the embedding of the *i*-th item.

### Multi-intent self-attention

Generally, the behavior of users is influenced by multiple intents, such as demand at the time or matching a specific interest. We now introduce the concept of multi-intent into the self-attention module to reflect user’s multiple intents in the recommendation field. The involvement of such domain knowledge is necessary and effective. Besides, the sparsity of interaction data leads to limited expressiveness of many learned item embeddings. It is better to pay more attention to learn the influential latent intents, which is useful for predicting the user’s next interaction. After all, it is the underlying intents that spawn a series of item interactions. Intents are easier to learn as they are usually correlated with many items, and richer information reduces the difficulty of learning.

In this module, we will maintain a set of global representation of latent intents and use them as the important mediator to upgrade the item embeddings in sequence. The self-attention module can be described as mapping a query and a set of key-value pairs to an output, we first get these vectors from the input using formula:

(5) $$\matrix{ Q \hfill & { = E{W_{q1}} + P{W_{q2}}} \hfill \cr }$$

(6) $$\matrix{ K \hfill & { = E{W_{k1}} + P{W_{k2}}} \hfill \cr }$$

(7) $$\matrix{ V \hfill & { = E{W_{v1}} + P{W_{v2}}} \hfill \cr }$$

where ${W_{q1}},{W_{q2}},{W_{k1}},{W_{k2}},{W_{v1}},{W_{v2}} \in {{\mathbb R}^{d \times d}}$ are trainable parameters. $P \in {{\mathbb R}^{n \times d}}$ is the positional encoding with all *n* time steps, which is added to preserve the influence of time order in the sequence.

### Multi-intent extractor

We adapt the self-attention module to extract multiple intents from the behavior sequence. Suppose users have at most *k* latent intents, which is usually a small number. We represent these intents with a learnable memory matrix $M = ({{\bf m}_1},...,{{\bf m}_k}) \in {{\mathbb R}^{k \times d}}$. Through *M*, we will obtain a new representation of the input sequence in the intent space, which we denote as function *g*. Given the input of this module $H \in {{\mathbb R}^{n \times d}}$, we first calculate the relevance score *C* between the latent intents and interacted items by the following formula:

(8) $$C = {\rm softmax}\big(M{H^{\rm \top }}\big)$$

where $C \in {{\mathbb R}^{k \times n}}$. The matrix *C* depicts *k* perspectives of the input sequence, and the weighted sum of item embeddings with coefficient matrix *C* can obtain *k* vector representations of the user to reflect the intents.

(9) $${M_s} = g(H) = CH$$

where ${M_s} \in {{\mathbb R}^{k \times d}}$. We now have completed the representation transformation of the sequence from the item space to the intent space. The updated representation will aggregate more useful information and thus be more expressive as the latent intents *M* can collect the user’ preferences contained in the behavior sequence from a global perspective. Next we show how to integrate the multi-intent feature into the self-attention mechanism to exploit its powerful representational capability.

### Multi-intent integration

With the above multi-intent extractor, we transform the two (*n* × *d*) dimensional key and value matrices *K* and *V* into the (*k* × *d*) dimensional intent space. Then, we computed the relationship between items in the sequence and their corresponding representations in the intent space. We finally use such relationship to reconstruct intent-aware item representations in the sequence:

(10) $$\matrix{ S \hfill & { = {\rm Attention}(Q,g(K),g(V))} \hfill \cr {} \hfill & {} \hfill \cr {} \hfill & { = {\rm softmax}\left( {\displaystyle{{Qg{{(K)}^T}} \over {\sqrt d }}} \right) \cdot g(V)} \hfill \cr }$$

where $S \in {{\mathbb R}^{n \times d}}$. So far, the upgrade of the traditional self-attention module is completed. Since the self-attention mechanism is well known, we will not introduce other modules in the mechanism that we do not modify, such as the position-wise feed-forward network.

Several self-attention blocks can be stacked to capture more complex transition patterns. Assuming the result of the entire self-attention encoder (SAE) is

(11) $${{ H}^L} = SAE \left( { E} \right)$$

where the last hidden vector ${ h}_t^L$ in ${{ H}^L} = \left[ {{ h}_0^L,{ h}_1^L, \ldots ,{ h}_t^L} \right]$ is selected to be the final extracted representation of the input behavior sequence.

### Recommendation learning

In order to make recommendations more accurate, we need to reduce the traditional ranking loss, which first measure distances between the sequence representation ${\bf h}_t^L$ and all items ***V*** by dot product:

(12) $$\widehat { y} = {\rm softmax} \left( {{ Vh}_t^L} \right)$$

where $\widehat { y} \in {{\mathbb R}^{|V|}}$. With the index of ground truth item converted into a one-hot vector ***y***, the cross-entropy loss is is adopted as:

(13) $${\ell _{{\rm Rec}}} = - {\rm one{-}hot}({{ y}_i})\log (\widehat {{{ y}_i}}).$$

### Multi-policy relay contrastive learning

Previous models have shown that data augmentations in CL-based sequential recommendation play a vital role in representation quality (Xie et al., 2020; Qiu et al., 2022). The commonly used data augmentations can mainly be divided into two types. The first is random data augmentation: item masking, item cropping and item reordering used in CL4Rec (Xie et al., 2020), which contrast sequences augmented from the same input. The second is semantic augmentation proposed in DuoRec (Qiu et al., 2022). It considers sequences having the same target item as semantically similar ones. Each type of the aforementioned data augmentations has its own advantage and exploits different self-supervision signals. A natural thought is whether it is possible to obtain comprehensive self-supervision by combining different data augmentations into one recommendation model. The methods in random data augmentation are relatively easy to coordinate as done in CL4SRec, which randomly samples two methods from the three data augmentation operators and applies them to the behavior sequence. Unfortunately, once the semantic augmentation method is introduced as done in CL4SRec, the complementary effect we expected did not appear, but instead performance degradation happened. We will demonstrate this phenomenon in the ablation study. Considering the above phenomenon, what we want is designing a feasible solution to achieve better performance when faced with data augmentation methods that are quite different and difficult to cooperate with each other.

To take full advantage of different data augmentation schemes in CL, especially those that are fundamentally different, we propose a multi-policy relay (MPR) training strategy. In order to avoid interference between different augmentation operators, MPR separates the training into multiple stages based on the number of data augmentations we will use. In each stage we relay optimize model parameters upon results from the previous stage. An example is shown in Fig. 3, where there are three data augmentation policies there: semantic augmentation, item masking augmentation and item cropping augmentation. Three stages are required to coordinate them together. In each stage, we leverage a multi-task learning strategy to jointly train the traditional ranking loss and the corresponding contrastive loss.

In-batch negative sampling is used to gather negative (dissimilar) samples for an augmented pair of samples, which means all the other augmented samples in a training batch are thought negative ones. Given a training batch $B = \left\{ {{{{ {h}^{\prime}}}_{\hskip-2pt 1}},{{{ {h}^{\prime}}}_{\hskip-2pt 2}}, \ldots ,{{{ {h}^{\prime}}}_{\hskip-2pt |B|}}} \right\}$ with the batch size |*B*|, there will be 2|*B*| hidden vectors after the augmentation, $\left\{ {{{{ {h}^{\prime}}_{\hskip-2pt 1}}},{{{ {h}^{\prime}}}_{{\hskip-2pt 1,s}}},{{{ {h}^{\prime}}}_{\hskip-2pt 2}},{{{ {h}^{\prime}}}_{\hskip-2pt 2,s}}, \ldots ,{{{ {h}^{\prime}}}_{\hskip-2pt |B|}},{{{ {h}^{\prime}}}_{\hskip-2pt |B|,s}}} \right\}$, where the subscripts denote the index in the batch and the augmentations for clarity. We can see that each positive pair in the batch is accompanied by 2(|*B*| − 1) negative pairs denoted as ${S^ - }$. For instance, for the augmented pair of sequence representations ${{ {h}^{\prime}}_{\hskip-2pt 1}}$ and ${{ {h}^{\prime}}_{\hskip-2pt 1,s}}$, the corresponding negative set is $S_1^ - = \left\{ {{{{ {h}^{\prime}}}_{\hskip-2pt 2}},{{{ {h}^{\prime}}}_{\hskip-2pt 2,s}},{{{ {h}^{\prime}}}_{\hskip-2pt 3}},{{{ {h}^{\prime}}}_{\hskip-2pt 3,s}}, \ldots ,{{{ {h}^{\prime}}}_{\hskip-2pt |B|}},{{{ {h}^{\prime}}}_{\hskip-2pt |B|,s}}} \right\}$. The contrastive loss for the batch *B* is the commonly used InfoNCE loss (Van den Oord, Li & Vinyals, 2018):

(14) $$\matrix{ {{l_{{\rm aug}}}} & { = \mathop {\rm {\mathbb E}}\limits_{i \in |B|} \left[ { - \log \displaystyle{{{e^{{{({{{ {h}^{\prime}}}_i})}^{\rm \top }}({{{ {h}^{\prime}}}_{i,s}})/\tau }}} \over {{e^{{{({{{ {h}^{\prime}}}_i})}^{\rm \top }}({{{ {h}^{\prime}}}_{i,s}})/\tau }} + \sum\nolimits_{{{\bf s}^ - } \in S_i^ - } {{e^{{{({{{ {h}^{\prime}}}_i})}^{\rm \top }}({{ s}^ - })/\tau }}} }}} \right]}. \cr }$$

Thus, the overall objective of MMRec with $\lambda$ scale weight is:

(15) $$\ell = {\ell _{{\rm Rec}}} + \lambda {\ell _{{\rm aug}}}.$$

### Model complexity analyses

In this section, we give a brief analysis of the complexity of the proposed MMRec. As our model modifies the self-attention mechanism to extract multiple latent intents from the user behavior sequences that represent different tastes of users, let us now look at the difference of complexity before and after the modification.

The complexity bottleneck in self-attention mechanism mainly comes from that each item’s vector representation is updated by integrating all other items in the behavior sequence, which is critical for retaining information far from the current position. However, considering all items triggers a complexity of $O({n^2})$ with respect to sequence length. The self-attention block is defined as:

(16) $${\rm Self{-}Attention}(Q,K,V) = {\rm softmax}\left( {\displaystyle{{Q{W_q}{{(K{W_k})}^T}} \over {\sqrt d }}} \right)V{W_v},$$

where *Q*, *K*, *V* are the input matrices, ${W_q},{W_k},{W_v} \in {{\mathbb R}^{d \times d}}$ are trainable parameters and *d* is the dimension of embedding. The cost of calculating the context aggregation matrix $Q{W_q}{(K{W_k})^T}$ is expensive. It needs multiplying two *n* × *d* matrices, which involves the complexity of $O({n^2})$ in time and space. In our multi-intent self-attention mechanism, the context aggregation matrix defined in Eq. (10) has the complexity $O(nk + {n^2}k)$, where *k* is usually much smaller than *n*. It can be seen that our model has paid a certain computational cost for integrating multi-intent into the self-attention mechanism, but it is worth as accuracy is what we care about most. We will see in the experimental part that the difference of training time is not large in many cases.

## Evaluation

To verify the effectiveness of the proposed MMRec, we perform an empirical evaluation for the recommendation task. In this section, we describe the experimental configuration and evaluate the performance of the proposed model.

### Datasets

We conduct extensive experiments on four public datasets collected from the real-world platforms, which can be accessed here (https://github.com/RuihongQiu/DuoRec). The statistics about them are summarized in Table 1.

**Table 1:** Statistics of datasets.

Dataset	Users	Items	Avg. length	Actions	Sparsity%
Beauty	22,363	12,101	8.9	198,502	99.93
Sports	35,598	18,357	8.3	296,337	99.95
Clothing	39,387	23,033	7.1	278,677	99.97
ML-1M	6,041	3,417	165.5	999,611	95.16

- **Amazon Beauty**, **Clothing** and **Sports** (McAuley et al., 2015) (http://jmcauley.ucsd.edu/data/amazon/). We select these three widely used Amazon dataset that vary in domain and scale.
- **MovieLens-1M (ML-1M)** (Harper & Konstan, 2015) (https://grouplens.org/datasets/movielens/1m/). A dataset of users rating movies is used here.

As done in previous methods (Qiu et al., 2022), data labels are binarized to be implicit feedback. To ensure data quality, users and items appearing less than five times are filtered out. The maximum length of a sequence is 50.

#### Metrics

We adopt two widely used Top-*K* metrics Hit Ratio (H@*K*) and Normalized Discounted Cumulative Gain (N@*K*) to evaluate the performance, where *K* is set to 5 and 10 for consistency. H@*K* represents the percentage that recommended items contain at least one ground truth item in top *K* position. N@*K* measures the specific ranking quality that assigns high scores to hit at top position ranks. We adopt the full-ranking strategy for fair comparison (Krichene & Rendle, 2020).

#### Baselines

We compare the proposed method with the following baseline methods.

- **BPR-MF** (Rendle et al., 2012) is the first method to use BPR loss to train a matrix factorization model.
- **Caser** (Tang & Wang, 2018) is a CNN-based method capturing high-order patterns by applying horizontal and vertical convolutional operations for sequential recommendation.
- **SASRec** (Kang & McAuley, 2018) utilizes self-attention to exploit the mutual influence between historical interactions. It is a strong baseline in sequential recommendation.
- **BERT4Rec** (Sun et al., 2019) learns a bidirectional representation model to make recommendations by learning a cloze objective with reference to BERT.
-
${{\bf S}^{\bf 3}}{\bf Rec}$ (Zhou et al., 2020) is a self-supervised learning framework under mutual information maximization principle. The Mask Item Prediction (MIP) variant is used here.
-
${\bf ComiRe}{{\rm c}_{{\bf CL}}}$ which combines the superior model in extracting multi-interest based on dynamic routing (Cen et al., 2020) and contrastive learning with tasks used the same as our MMRec.
- **CL4SRec** (Xie et al., 2020) uses item cropping, item masking, and item reordering as data augmentations for contrastive learning.
- **ICLRec** (Chen et al., 2022) learns users’ latent intents from the behavior sequences through clustering. It then integrates the learnt intents into the model *via* an auxiliary contrastive loss.
- **DuoRec** (Qiu et al., 2022) uses semantic similarity as a robust data augmentation. In this scenario sequences which have the same target item are positive pairs.

#### Implementation

We implement the proposed model with RecBole (https://github.com/RUCAIBox/RecBole), which is a unified open-source framework to develop and reproduce recommendation algorithms. The embedding size is set to 64. The numbers of layers and heads in the self-attention mechanism are set to 2. The dropout (Srivastava et al., 2014) rate are chosen from {0.1, 0.2, 0.3, 0.4, 0.5}. The batch size is set to 256. We use the Adam (Kingma & Ba, 2014) optimizer with the initial learning rate 0.001. $\lambda$ in Eq. (15) is chosen from {0.1, 0.2, 0.3, 0.4, 0.5}. We tested the crop/mask proportion of items from 0.1 to 0.9, and the number of intents from 11 to 39.

### Overall results

Table 2 presents the performance comparison between several baselines and the proposed MMRec, and we have the following observations.

**Table 2:** Overall performance.

Dataset	Metric	BPR-MF	Caser	SASRec	BERT4Rec	${{\rm S}^3}{\rm Rec}$	${\rm ComiRe}{{\rm c}_{{\rm CL}}}$	CL4SRec	ICLRec	DuoRec	MMRec	Improv (%)
Beauty	H@5	0.0120	0.0259	0.0365	0.0193	0.0327	0.0383	0.0401	0.0465	0.0543	**0.0607 **± 0.0011	11.8
	H@10	0.0299	0.0418	0.0627	0.0401	0.0591	0.0644	0.0683	0.0846	0.0834	**0.0889** ± 0.0019	5.1
	N@5	0.0040	0.0127	0.0236	0.0187	0.0175	0.0228	0.0223	0.0256	0.0343	**0.0378** ± 0.0008	10.2
	N@10	0.0053	0.0253	0.0281	0.0254	0.0268	0.0292	0.0317	0.0355	0.0437	**0.0468** ± 0.0017	7.1
Clothing	H@5	0.0067	0.0108	0.0168	0.0125	0.0163	0.0159	0.0168	0.0178	0.0192	**0.0204** ± 0.0010	6.25
	H@10	0.0094	0.0174	0.0272	0.0208	0.0237	0.0271	0.0266	0.0279	0.0296	**0.0314** ± 0.0017	6.08
	N@5	0.0052	0.0067	0.0091	0.0075	0.0101	0.0095	0.0090	0.0097	0.0110	**0.0117** ± 0.0008	6.36
	N@10	0.0069	0.0098	0.0124	0.0102	0.0132	0.0130	0.0121	0.0129	0.0144	**0.0153** ± 0.0015	6.25
Sports	H@5	0.0092	0.0139	0.0218	0.0176	0.0157	0.0221	0.0227	0.0295	0.0307	**0.0321** ± 0.0007	4.56
	H@10	0.0188	0.0231	0.0336	0.0326	0.0265	0.0351	0.0374	0.0441	0.0472	**0.0485** ± 0.0015	2.76
	N@5	0.0040	0.0085	0.0127	0.0105	0.0098	0.0118	0.0129	0.0171	0.0191	**0.0200** ± 0.0009	4.72
	N@10	0.0051	0.0126	0.0169	0.0153	0.0135	0.0177	0.0184	0.0237	0.0244	**0.0252** ± 0.0011	3.28
ML-1M	H@5	0.0078	0.0816	0.1087	0.0733	0.1078	0.1233	0.1147	0.1250	0.1632	**0.1784** ± 0.0029	9.4
	H@10	0.0162	0.1593	0.1904	0.1323	0.1952	0.2072	0.1975	0.2113	0.2485	**0.2651** ± 0.0041	6.7
	N@5	0.0052	0.0372	0.0638	0.0432	0.0616	0.0719	0.0662	0.0714	0.1046	**0.1176** ± 0.0025	12.5
	N@10	0.0079	0.0624	0.0910	0.0619	0.0917	0.0998	0.0928	0.0992	0.1321	**0.1455** ± 0.0037	10.2

**Note:**

The best result is bolded and the runner-up is underlined. The MMRec achieves the state-of-the-art result among all baseline models.

- Sequential models like Caser behave better than the traditional collaborative filtering method BPR-MF. This is due to the modeling of sequential dependency. Models like SASRec and BERT4Rec have achieved extraordinary results for their good mechanisms. But they contain a large number of parameters, which requires a lot of data feeding to get a good model. This limits their potential and can be a problem for sparse datasets.
- The performance of CL-based sequential models like CL4SRec, ICLRec and DuoRec are generally better than traditional sequential recommendation methods. This demonstrates the benefits of introducing contrastive learning framework to mine intrinsic information hidden in the sequence. Moreover, we can observe that the improvement brought by capturing user’s intents with ${\rm ComiRe}{{\rm c}_{{\rm CL}}}$ is more significant on ML-1M dataset. This shows that dynamic routing suits more on denser dataset. With regard to DuoRec, its performance is impressive, which demonstrates the effectiveness of augmenting sequences that have the same target item as similar pairs.
- Finally, we can see that our proposed MMRec consistently outperforms other competitive methods, which clearly proves the correctness of the model design. The advantage is brought by adapting the self-attention backbone network to integrate user’s latent intents, as well as the multi-policy relay training strategy to maximize the complementary effects of multiple contrast tasks that have very different underlying mechanisms. We can notice that the improvements on two datasets Beauty and ML-1M, which have longer average length, are the most. This is because longer sequence generally has a larger number of latent intents, and the auxiliary multi-intent feature can play a greater role in this scenario.

### Ablation study

#### The impact of multi-policy relay contrastive learning

**Figure 4:**
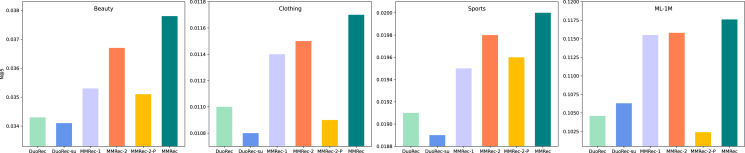
The performance comparison with different variants on four datasets (N@5).

It is still not clear how important the mechanism of Multi-Policy Relay Contrastive Learning is. We now explore this question with some ablation experiments to reveal it. The proposed MMRec selects three data augmentation methods in this mechanism, which are semantic augmentation, item masking and item reordering in order of execution. We compare two variants of MMRec: MMRec-1 that reserves only semantic augmentation as the data augmentation method and MMRec-2 that reserves both semantic augmentation and item masking as the way of data augmentation in this mechanism. We report the performance on four datasets in Table 3. Compared with the one policy model MMRec-1, we observe that the MMRec-2 with two policies and MMRec with three policies can both improve the performance effectively. The gains from different data augmentation policies vary on different datasets. However, combining multiple policies according to our mechanism achieves the best performance. The average improvements of this mechanism on the four datasets are 7.6%, 4.2%, 4.3% and 1.2% respectively. In order to have an intuitive comparison of their gaps, we also draw the results with respect to N@5 in Fig. 4.

**Table 3:** Ablation study with different variants of MMRec.

Methods	Beauty		Clothing		Sports		ML-1M	
	H@5	N@5	H@5	N@5	H@5	N@5	H@5	N@5
DuoRec	0.0543	0.0343	0.0192	0.0110	0.0307	0.0191	0.1632	0.1046
DuoRec-su	0.0545	0.0341	0.0190	0.0108	0.0302	0.0189	0.1657	0.1063
MMRec-1	0.0562	0.0353	0.0193	0.0114	0.0304	0.0195	0.1774	0.1155
MMRec-2	0.0590	0.0367	0.0195	0.0115	0.0316	0.0198	0.1772	0.1158
MMRec-2-P	0.0549	0.0351	0.0183	0.0109	0.0307	0.0196	0.1594	0.1024
MMRec	0.0607	0.0378	0.0204	0.0117	0.0321	0.0200	0.1784	0.1176

Let’s see what happens if we change from the serial mode to parallel mode, *i.e.*, add up the contrastive losses of both tasks to train them at the same time as done in CL4SRec. The corresponding results are recorded in the row named MMRec-2-P. Significant performance degradation can be observed compared to results of the MMRec-2 which follows our strategy of Multi-Policy Relay. This fully shows the necessity and superiority of this mechanism when combining multiple fundamentally different data augmentation operators.

Two kinds of augmentations are proposed in DuoRec (Qiu et al., 2022), one is the model-level augmentation based on Dropout and another is the semantic augmentation adopted in our model. We should note here that only the second belongs to the traditional data augmentations in contrastive learning. We show the results of DuoRec removing the model-level augmentation named DuoRec-su in Table 3. The results show that the effect of model-level augmentation is limited, and even has a negative effect on dataset ML-1M. This is why there is no policy of model-level augmentation in our Multi-Policy Relay Contrastive Learning. Compared with DuoRec-su, the extra contrastive task of item mask in MMRec-2-P did not help it gain any advantage, a slight disadvantage is observed on the contrary. This again shows that some tasks with large differences in mechanism cannot cooperate well. This is where our strategy can play a bigger role.

#### The impact of multi-intent self-attention mechanism

**Figure 5:**
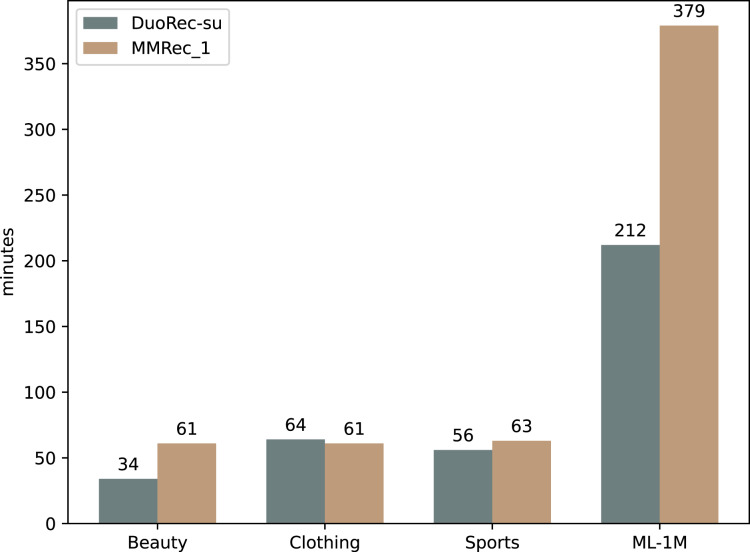
The comparison of time cost brought by the integration of multi-intent in the self-attention mechanism. Time is measured in minutes.

We further investigate the role of multi-intent self-attention mechanism by some experiments. DuoRec-su has the traditional self-attention mechanism as the backbone network, and the accompanying contrast task is semantic augmentation. The only difference between DuoRec-su and MMRec-1 is that the backbone network in MMRec-1 is replaced by our multi-intent self-attention network. The comparison between them demonstrates the importance of introducing multi-intent into the self-attention module to reflect user’s multiple intents in practice and mitigate the data sparsity. The average improvements of this mechanism on the four datasets are 3.4%, 3.6%, 1.9% and 7.9% respectively. Figure 4 shows a more intuitive comparison. The additional time cost brought by the integration of this multi-intent mechanism is demonstrated in Fig. 5. We can find that the difference in training time is not large on most datasets, except the ML-1M. This is because the average sequence length of ML-1M is relatively large, resulting in the need for a larger k when extracting intents. We have shown in the “Model Complexity Analyses” section that there is a linear relationship between the training time and the k value. The problem of data sparsity limits the expressiveness of the learned embeddings of many items. And shifting the representation learning from all items to more relevant and focused intents will allow us to obtain stronger predictive power.

#### The impact of the number of interests k

**Figure 6:**
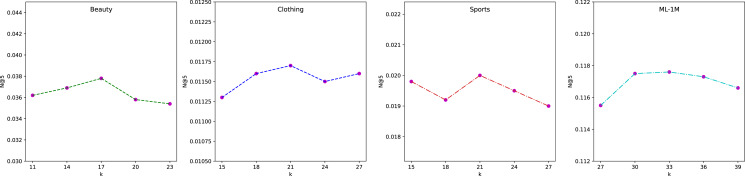
Effectiveness of the intents number *k* on four datasets (N@5).

To study the influence of intent number, we vary k and demonstrate the performance comparison with respect to N@5 on four datasets in Fig. 6. We observe that different datasets have their own suitable range of values. The best results can be achieved by discovering their optimal interval. From the results, we can find that within the optimal interval in each dataset, the fluctuations are small, which validates the stability of our method. Besides, compared with other datasets, the optimal k is the largest for ML-1M because it has the longest average sequence length.

#### Visualization of intents

**Figure 7:**
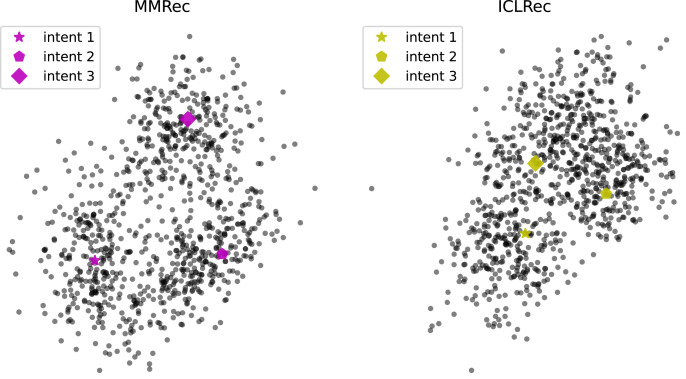
Visualization of a portion of intents and items’ representations *via* t-SNE for MMRec and *ICLRec* on the dataset Beauty.

To better understand how intents benefit prediction of model, we visualize the learned intents and items’ representations *via* t-SNE (Van der Maaten & Hinton, 2008), which is used to reveal structure of high-dimensional data on low-dimensional manifolds as we cannot clearly plot more than two dimensions. Specifically, we select three most item-centric intents and sampled 300 items surrounding them. Figure 7 shows the results of applying t-SNE for MMRec and *ICLRec* on dataset Beauty. Based on the trend depicted in Fig. 7, we can intuit that an intent is like the center of a cluster, and related items are clustered around them, which gives the model clearer grasp of the purpose of items. This may be the reason why the introducing of intents can promote model’s performance. Besides, we can observe that compared with *ICLRec*, MMRec shows more obvious distinguishability. This explains why our model is better than *ICLRec*.

## Conclusion

In this work, we improved sequential recommendation with contrastive learning using a novel model named MMRec. Compared with previous models, MMRec mainly has two main innovations. First, we introduced latent intents into the self-attention module, which is commonly adopted by the current state of the art models as the backbone network. This is more in-line with the characteristics of behavior sequences in recommendation scenarios. Datasets in this field are often sparse, and the representation of many items are destined to be learned poorly. It will be more reasonable to learn a few key latent intents that have larger influence on the sequence evolution. Second, there are some data augmentations that are very different in contrastive learning tasks. Significant differences in their internal mechanisms makes them difficult to coordinate. Our proposed mechanism of Multi-Policy Relay Contrastive Learning can reduce the interference by separating the model training into multiple stages where each stage corresponds to one data augmentation method in the contrastive learning task. Experimental results and analysis demonstrate the superiority of our proposed model.

## Supplemental Information

10.7717/peerj-cs.1088/supp-1 Supplemental Information 1 Source code to run the proposed model MMRec.

## Additional Information and Declarations

### Data Availability

The following information was supplied regarding data availability: The code is available in the Supplemental File. The data is available at GitHub: https://github.com/RuihongQiu/DuoRec.

## References

[ref-1] Cen Y, Zhang J, Zou X, Zhou C, Yang H, Tang J (2020). Controllable multi-interest framework for recommendation. ArXiv preprint.

[ref-2] Chen Y, Liu Z, Li J, McAuley J, Xiong C (2022). Intent contrastive learning for sequential recommendation.

[ref-3] Devlin J, Chang M-W, Lee K, Toutanova K (2018). Bert: pre-training of deep bidirectional transformers for language understanding. ArXiv preprint.

[ref-4] Ding K, Xu Z, Tong H, Liu H (2022). Data augmentation for deep graph learning: a survey. ArXiv preprint.

[ref-5] Gutmann MU, Hyvärinen A (2012). Noise-contrastive estimation of unnormalized statistical models, with applications to natural image statistics. Journal of Machine Learning Research.

[ref-6] Harper FM, Konstan JA (2015). The MovieLens datasets: history and context. ACM Transactions on Interactive Intelligent Systems.

[ref-7] He Z, Zhao H, Lin Z, Wang Z, Kale A, Mcauley J (2021). Locker: locally constrained self-attentive sequential recommendation.

[ref-8] Hidasi B, Karatzoglou A, Baltrunas L, Tikk D (2015). Session-based recommendations with recurrent neural networks. ArXiv preprint.

[ref-9] Hjelm RD, Fedorov A, Lavoie-Marchildon S, Grewal K, Bachman P, Trischler A, Bengio Y (2018). Learning deep representations by mutual information estimation and maximization. ArXiv preprint.

[ref-10] Jaiswal A, Babu AR, Zadeh MZ, Banerjee D, Makedon F (2021). A survey on contrastive self-supervised learning. Technologies.

[ref-11] Kang W-C, McAuley J (2018). Self-attentive sequential recommendation.

[ref-12] Kingma DP, Ba J (2014). Adam: a method for stochastic optimization. ArXiv preprint.

[ref-13] Koren Y, Bell R, Volinsky C (2009). Matrix factorization techniques for recommender systems. Computer.

[ref-14] Krichene W, Rendle S (2020). On sampled metrics for item recommendation.

[ref-15] Li B, Hou Y, Che W (2022). Data augmentation approaches in natural language processing: a survey. AI Open.

[ref-16] Li C, Liu Z, Wu M, Xu Y, Zhao H, Huang P, Kang G, Chen Q, Li W, Lee DL (2019). Multi-interest network with dynamic routing for recommendation at Tmall.

[ref-17] Li H, Wang X, Zhang Z, Ma J, Cui P, Zhu W (2021). Intention-aware sequential recommendation with structured intent transition. IEEE Transactions on Knowledge and Data Engineering.

[ref-18] Liu Z, Li X, Fan Z, Guo S, Achan K, Philip SY (2020). Basket recommendation with multi-intent translation graph neural network.

[ref-19] Liu X, Zhang F, Hou Z, Mian L, Wang Z, Zhang J, Tang J (2021). Self-supervised learning: generative or contrastive. IEEE Transactions on Knowledge and Data Engineering.

[ref-20] Lv F, Jin T, Yu C, Sun F, Lin Q, Yang K, Ng W (2019). SDM: sequential deep matching model for online large-scale recommender system.

[ref-21] Ma C, Kang P, Liu X (2019). Hierarchical gating networks for sequential recommendation.

[ref-22] Ma J, Zhou C, Yang H, Cui P, Wang X, Zhu W (2020). Disentangled self-supervision in sequential recommenders.

[ref-23] McAuley J, Targett C, Shi Q, Van Den Hengel A (2015). Image-based recommendations on styles and substitutes.

[ref-24] Mei L, Ren P, Chen Z, Nie L, Ma J, Nie J-Y (2018). An attentive interaction network for context-aware recommendations.

[ref-25] Pan Z, Cai F, Ling Y, de Rijke M (2020). An intent-guided collaborative machine for session-based recommendation.

[ref-26] Pei W, Yang J, Sun Z, Zhang J, Bozzon A, Tax DM (2017). Interacting attention-gated recurrent networks for recommendation.

[ref-27] Qiu R, Huang Z, Yin H, Wang Z (2022). Contrastive learning for representation degeneration problem in sequential recommendation.

[ref-28] Rendle S, Freudenthaler C, Gantner Z, Schmidt-Thieme L (2012). BPR: Bayesian personalized ranking from implicit feedback. ArXiv preprint.

[ref-29] Rendle S, Freudenthaler C, Schmidt-Thieme L (2010). Factorizing personalized Markov chains for next-basket recommendation.

[ref-30] Sabour S, Frosst N, Hinton GE (2017). Dynamic routing between capsules.

[ref-31] Srivastava N, Hinton G, Krizhevsky A, Sutskever I, Salakhutdinov R (2014). Dropout: a simple way to prevent neural networks from overfitting. The Journal of Machine Learning Research.

[ref-32] Sun F, Liu J, Wu J, Pei C, Lin X, Ou W, Jiang P (2019). BERT4Rec: sequential recommendation with bidirectional encoder representations from transformer.

[ref-33] Tang J, Wang K (2018). Personalized Top-N sequential recommendation via convolutional sequence embedding.

[ref-34] Tanjim MM, Su C, Benjamin E, Hu D, Hong L, McAuley J (2020). Attentive sequential models of latent intent for next item recommendation.

[ref-35] Tian Y, Sun C, Poole B, Krishnan D, Schmid C, Isola P (2020). What makes for good views for contrastive learning?. Advances in Neural Information Processing Systems.

[ref-36] Van den Oord A, Li Y, Vinyals O (2018). Representation learning with contrastive predictive coding. ArXiv e-prints.

[ref-37] Van der Maaten L, Hinton G (2008). Visualizing data using t-SNE. Journal of Machine Learning Research.

[ref-38] Vaswani A, Shazeer N, Parmar N, Uszkoreit J, Jones L, Gomez AN, Kaiser Ł, Polosukhin I (2017). Attention is all you need.

[ref-39] Xie X, Sun F, Liu Z, Wu S, Gao J, Ding B, Cui B (2020). Contrastive learning for sequential recommendation. ArXiv preprint.

[ref-40] Ying H, Zhuang F, Zhang F, Liu Y, Xu G, Xie X, Xiong H, Wu J (2018). Sequential recommender system based on hierarchical attention network.

[ref-41] Yuan F, Karatzoglou A, Arapakis I, Jose JM, He X (2019). A simple convolutional generative network for next item recommendation.

[ref-42] Zhou K, Wang H, Zhao WX, Zhu Y, Wang S, Zhang F, Wang Z, Wen J-R (2020). S3-Rec: self-supervised learning for sequential recommendation with mutual information maximization.

[ref-43] Zimdars A, Chickering DM, Meek C (2013). Using temporal data for making recommendations. ArXiv preprint.

